# Cognitive and Emotional Regulation for High-Performance Poker Players: A Multidisciplinary Therapeutic Approach

**DOI:** 10.1192/j.eurpsy.2025.2154

**Published:** 2025-08-26

**Authors:** M. Ribeiro, E. de Lanna, L. S. Ribeiro

**Affiliations:** 1 Private Practice, Delray Beach, United States; 2 Psychology, Universidade Federal Fluminense, Volta Redonda; 3 Psychology, Pontifical Catholic University of Rio de Janeiro; 4 Psychology, Private Practice, Rio de Janeiro, Brazil

## Abstract

**Introduction:**

High-performance players in sports, poker, or other competitive fields share traits that support them to excel under pressure. Exceptional players show mental resilience, staying focused, and adaptability skills in high-stress situations. However, many players struggle with consistency and discipline when emotional influence occurs over their routine and decision-making on tables. This work addresses common challenges for high-performance poker players and explores therapeutic techniques that improve decision-making and overall well-being.

**Objectives:**

High-performance poker players, particularly cash game players, encounter unique cognitive and emotional challenges due to high-stakes, financial volatility, and emotional dysregulation. This work addresses common therapeutic demands and multidisciplinary approaches to support high-performance poker players.

**Methods:**

Players who received therapeutic support benefited from a combination of cognitive-behavioral strategies and positive psychology, particularly focusing on emotional regulation and goal-setting to enhance their sense of control, motivation, decision-making, psychological well-being, and overall performance. The therapeutic interventions specifically targeted cognitive distortions and behavioral patterns that undermine decision-making and contribute to emotional dysregulation. Techniques such as cognitive restructuring, mindfulness, and problem-solving skills training through deliberate practice, promoting practical tools to manage the unique cognitive and emotional challenges faced in poker.

**Results:**

The findings show that integrating cognitive-behavioral techniques into daily routines improves consistency, making daily strategies more automatic. Mental exercises like mindfulness and cognitive reframing strengthened skills while monitoring emotional states and helped players detect early signs of “tilt” and regulate emotions, diminishing impulsive decisions and heightening emotional responses. Addressing cognitive distortions, such as confirmation bias, through journaling and stress management techniques like deep breathing, helped players maintain composure under pressure. Therefore, these approaches assisted poker players in improving their adaptability skills without sacrificing consistency, leading to better performance and long-term success.

**Conclusions:**

The cognitive and emotional demands of high-stakes poker require a comprehensive mental regulation approach. Combining cognitive-behavioral techniques, psychodynamic strategies, and mindfulness improves emotional stability, decision-making, and performance. Addressing cognitive distortions and using emotional regulation techniques help players avoid emotionally-driven decisions and maintain focus under pressure. By integrating these strategies into their routines, poker players can enhance consistency, optimize cognitive function, and achieve long-term success.

**Disclosure of Interest:**

None Declared

